# GDI-Mediated Cell Polarization in Yeast Provides Precise Spatial and Temporal Control of Cdc42 Signaling

**DOI:** 10.1371/journal.pcbi.1003396

**Published:** 2013-12-12

**Authors:** Ben Klünder, Tina Freisinger, Roland Wedlich-Söldner, Erwin Frey

**Affiliations:** 1 Arnold Sommerfeld Center for Theoretical Physics (ASC) and Center for NanoScience (CeNS), Department of Physics, Ludwig-Maximilians-Universität München, München, Germany; 2 Max Planck Institute of Biochemistry, Cellular Dynamics and Cell Patterning, Martinsried, Germany; University of Virginia, United States of America

## Abstract

Cell polarization is a prerequisite for essential processes such as cell migration, proliferation or differentiation. The yeast *Saccharomyces cerevisiae* under control of the GTPase Cdc42 is able to polarize without the help of cytoskeletal structures and spatial cues through a pathway depending on its guanine nucleotide dissociation inhibitor (GDI) Rdi1. To develop a fundamental understanding of yeast polarization we establish a detailed mechanistic model of GDI-mediated polarization. We show that GDI-mediated polarization provides precise spatial and temporal control of Cdc42 signaling and give experimental evidence for our findings. Cell cycle induced changes of Cdc42 regulation enhance positive feedback loops of active Cdc42 production, and thereby allow simultaneous switch-like regulation of focused polarity and Cdc42 activation. This regulation drives the direct formation of a unique polarity cluster with characteristic narrowing dynamics, as opposed to the previously proposed competition between transient clusters. As the key components of the studied system are conserved among eukaryotes, we expect our findings also to apply to cell polarization in other organisms.

## Author Summary

Cell polarization is a fundamental cellular process that defines a single orientation axis within prokaryotic or eukaryotic cells and is a prerequisite for developmental processes such as cell migration, proliferation or differentiation. In the yeast *Saccharomyces cerevisiae* cell polarization determines the position of a new growth or bud site. Although many studies have focused on identifying polarity regulators and their interactions, the fundamental mechanisms and features of cell polarity still remain controversial. Here, we develop a detailed mathematical model of diffusion-driven cell polarization, which we verify experimentally. We show that this polarization mechanism provides precise spatial and temporal control of signals, which determine the place of a new growth site. Changes induced by the cell cycle allow simultaneous switch-like regulation of polarization and activation of the GTPase Cdc42, the central polarity regulator which initiates formation of a new bud. This regulation drives direct formation of a unique Cdc42 cluster with characteristic narrowing dynamics and robustly narrow spatial focus. Hence, our analysis reveals fundamental design principles that allow cell polarization to reliably initiate developmental processes at a specific time and place. As the key components of the studied system are conserved among eukaryotes, we expect our findings also to apply to cell polarization in other organisms.

## Introduction

Establishment of cell polarity is a fundamental cellular process that defines orientation axes within prokaryotic or eukaryotic cells and is often referred to as ‘symmetry breaking’. Polarity axes are a prerequisite for many developmental and pathogenic processes such as cell migration, maintenance of epithelial tissue integrity, asymmetric stem cell division, or tumor development [1], [2], [3].

In the yeast *Saccharomyces cerevisiae*, the GTPase Cdc42 regulates cell polarization to determine the position of a new growth or bud site. A collection of different proteins under control of Cdc42 accumulates within a restricted region of the plasma membrane to initiate morphogenetic downstream events at the desired position. These clusters arise even in the absence of spatial cues [4], and are characterized by a dynamic equilibrium where clusters remain stable although individual proteins rapidly exchange between plasma membrane and cytoplasm [5]. Two pathways for Cdc42 localization have been identified that independently generate cell polarization [4], [5]. One pathway involves targeted exocytosis of membrane-bound Cdc42 along actin cables [6], [7], while the other one relies on fast recycling of Cdc42 through the cytosol by the guanine nucleotide dissociation inhibitor (GDI) Rdi1 [8]. Constitutively active or inactive Cdc42 cannot polarize without actin [5] indicating that the ability of Cdc42 to cycle between its active GTP-bound and inactive GDP-bound state is crucial for actin-independent polarization. Moreover, polarization has been shown to rely on a positive feedback loop of Cdc42-GTP recruiting its activator, the guanine nucleotide exchange factor (GEF) Cdc24, to the membrane [9], [10], [4], [11]. Although many studies have focused on identifying polarity regulators and their interactions, the fundamental mechanisms responsible for spontaneous polarization still remain controversial. Altschuler and colleagues put forward a conceptual model for yeast polarity establishment relying on a single positive feedback loop of a polarity protein locally enhancing its own membrane attachment [12]. The model predicts stochastic unstable polarization with reduced polarization efficiency at higher particle numbers where stochastic effects become smaller. This prediction was questioned by experiments showing that expression levels of Cdc42 did not influence polarization efficiency [13], [14]. In a more mechanistic approach Goryachev and Pokhilko developed a mathematical model for cell polarization in yeast, including putative molecular interactions for which experimental evidence is lacking [15], [14]. The proposed Turing-type mechanism for GDI-mediated polarization actively produces several macroscopic transient clusters upon polarization that then merge into a single cluster due to competition for limited amounts of proteins. Consistent with this prediction, multiple transient caps could be detected in a small subpopulation of wild-type cells [16], [13]. The model of Goryachev and Pokhilko was in subsequent studies extended or readjusted [16], [17], [18]. However, in a recent study we showed that formation of multiple stable polarization clusters depends on actin, and that GDI-mediated polarization rather counteracts the formation of these multiple clusters as long as it generates a single polarization site before actin structures have formed [14]. Given these findings it remains unclear at present how the GDI- and actin-dependent polarization mechanisms polarize independently, and how they contribute to the wild-type polarization dynamics. In addition, the role of the cell cycle in polarity regulation and the features of this regulation remain incompletely understood.

The coexistence of two independent polarization pathways with unresolved interactions and unclear polarization dynamics prompted us to investigate the fundamental features of GDI-mediated polarization in more molecular detail, and to address how polarity is established at first. Going beyond our previous work [14] we developed a detailed mechanistic model for the GDI pathway including a full three-dimensional description of the cell geometry as well as all relevant protein species and biochemical reactions, which then enabled us to identify characteristic properties of this polarization mechanism. Our model differs from other previous models in several critical aspects. Importantly, the core feedback underlying cell polarization in our model differs from those used previously, and all reactions and parameters in our mechanistic model are backed up by experiments. The ensuing predictions for the polarization dynamics are fundamentally different to previous models. Our new results provide strong evidence that GDI-mediated polarization is mainly driven by deterministic reaction-diffusion dynamics. The model provides a unified and comprehensive understanding of known mutant phenotypes and predicts several so far unknown phenotypes, which we could verify experimentally. In addition, we identify the enhancement of positive feedback loops in Cdc42 activation and recruitment as the molecular mechanism, which facilitates cell cycle control of GDI-mediated polarization. A detailed analysis of the polarization dynamics as well as systematic parameter variations reveal that GDI-mediated polarization provides precise spatial and temporal control of Cdc42-GTP production. Taken together, our combined theoretical and experimental analysis reveals the fundamental design principles that allow GDI-mediated cell polarization to reliably initiate developmental processes at a specific time and place.

## Results

**Figure 1:**
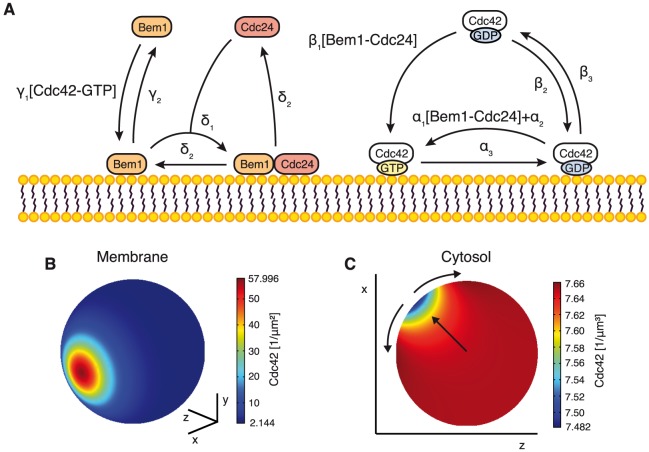
Details of the mechanistic model for GDI-mediated cell polarization in yeast. **A** Schematic representation of model reactions. A list of all model reactions is given in the Materials and Methods section. **B** Numerically obtained polarized Cdc42 distribution on the membrane for control conditions. **C** Corresponding cytosolic distribution of Cdc42 in the x-z plane. Arrows indicate protein flux.

For our theoretical analysis we first developed a detailed mechanistic model of GDI-mediated polarization. We considered a yeast cell as spherically-shaped cytosolic volume surrounded by a plasma membrane boundary. Proteins were allowed to either attach to the inner face of the plasma membrane or to remain in the soluble cytosolic pool. Cdc42, its GEF Cdc24 and effector Bem1 have been shown to act together to locally amplify the activation and accumulation of Cdc42 [9], [10]. We therefore explicitly included the concentrations and spatial distributions of these polarity proteins as model variables. We allowed the peripheral membrane proteins Cdc42, Cdc24, and Bem1 to freely diffuse with a diffusion constant of D_2_ = 0.03 µm^2^/s on the membrane as measured for Cdc42 and prenylated GFP [19], and with a diffusion constant of D_3_ = 11 µm^2^/s in the cytosol as measured for cytosolic GFP [20]. To estimate the amount of cellular Cdc42 we took into account that GDIs and GTPases form stoichiometric complexes in the cytosol [21]. With an average number of 1650 molecules of the yeast Rho GDI per cell [22] and a cytosolic fraction of Cdc42 of roughly 50% [5] we estimated the total cellular amount of Cdc42 to *N_42_* = 3000. Note that previous modeling approaches may have used an unrealistically high estimate of Cdc42 concentration derived from cultured mammalian cells [15], [17], [23]. We determined the average cell radius for G1-arrested cells used in this study to *R* = 3.95±0.05 µm (n = 63) and used the previously determined protein numbers per cell [22] of Bem1 (*N_B_* = 6500) and Cdc24 (*N_24_* = 1000). A schematic representation of the model reactions discussed below is shown in Figure 1A.

While Cdc42 is anchored to the membrane via a prenylation site and polybasic region irrespective of the bound nucleotide, extraction by the GDI preferentially occurs for the inactive GDP-bound form [24], [25], [14], which we implemented in our model with rate *β_3_*. Next we included a set of reactions describing a positive feedback loop recruiting the GEF Cdc24 towards Cdc42-GTP [9], [10]. Details of this feedback loop still remain controversial [11], [26], [27]. However, Bem1 and p21-activated kinase Cla4 likely contribute to the feedback loop as they interact with Cdc42, Cdc24 and each other [28], [9], [29]. Thus one would generically expect the Cdc24 concentration on the membrane to be proportional to the amount of Cdc42-GTP at the respective site. However, a previous modeling approach relied on a Cdc24 distribution with an effectively quadratic dependence on Cdc42-GTP [15], which would result in a more focused Cdc24 distribution compared to Cdc42-GTP on the membrane. Yet, Cdc24 was not found to be more focused compared to Cdc42 or other polarity regulators [8]. In addition, the model of Goryachev and Pokhilko predicts that increased Cdc42 activity would lead to slower Cdc24 turnover in polarity clusters, which we could also not confirm [14]. We therefore implemented the positive feedback loop by including a Bem1-mediated recruitment of Cdc24 towards active Cdc42 (Figure 1A): Cytosolic Bem1 attaches to the membrane with a rate equal to the local Cdc42-GTP concentration times a constant *γ_1_*. This reaction effectively describes targeting of Bem1 to the membrane by interaction with Cdc42-GTP or other Cdc42-GTP-bound proteins such as Cla4 [9], [10], [11] and subsequent binding of Bem1 to the membrane using its PX domain [11]. Cytosolic Cdc24 then binds to membrane-bound Bem1 with a rate *δ_1_* and forms a complex on the membrane; this accounts for the observation that Bem1 recruits Cdc24 to the membrane [9], [10], activates it [30] and maintains it there [31]. The corresponding reverse detachment reactions of Bem1 and Cdc24 are included with rates *γ_2_* and *δ_2_*, respectively, as both proteins rapidly exchange between polarity cluster and cytosol [5]. We implemented hydrolysis of GTP on Cdc42 by GTPase-activating proteins (GAPs) [32], [33], [34], [35] with a constant rate α_3_. Nucleotide-exchange of membrane-bound Cdc42-GDP takes place with a constant intrinsic rate *α_2_*, and in addition with a rate proportional to the local concentration of membrane bound Cdc24 times a constant *α_1_*, representing GEF-catalyzed nucleotide-exchange [32]. Cytosolic Cdc42-GDP attaches to the membrane with a low constant background rate *β_2_* [25]. Moreover, it attaches in GTP-bound form with a rate equal to the local membrane-bound Cdc24 concentration times a constant *β_1_*. We included the latter reaction to effectively describe a GEF-mediated displacement of Cdc42 from its GDI Rdi1 and subsequent nucleotide-exchange based on experimental evidence of GEF-mediated displacement of the GTPases Rac1 and Rab from their GDI [21], [36], [14]. A competition between GEF and GDI is also supported by the lack of membrane extraction when observing the dominant-negative Cdc42^D57Y^ mutant, which is supposed to sequester its GEF [37], [38], [14]. To conclude, the key mechanism of our model is a Cdc24-mediated positive feedback loop of Cdc42-GTP locally enhancing the activation and recruitment of additional Cdc42. A detailed description of all model reaction rates is given in the Materials and Methods section.

Mathematically, the spatio-temporal protein dynamics in the plasma membrane and the cytosol resulting from a combination of all above reactions and protein diffusion can be cast in the form of a set of partial differential equations (Materials and Methods). We employed analytical as well as numerical methods to solve these equations and thereby quantitatively determined the polarization efficiency and cluster dynamics for different genetic backgrounds. Importantly, we included a full three-dimensional description of membrane and cytosol to avoid approximations which qualitatively affect our conclusions on the dynamics (see Text S1).

### Stable cell polarization with continuous exchange of proteins

Initially we set out to characterize the properties of control cells. To this end we numerically simulated the protein dynamics starting from an unpolarized state and found that the system was able to efficiently evolve into a polarized steady state. Cdc42, Bem1 and Cdc24 accumulate in a cluster on the plasma membrane while the corresponding cytosolic concentrations remain almost homogeneous due to the rapid cytosolic diffusion similar to previous polarity models (e.g. [39]). An example for the simulated spatial Cdc42 distribution in control cells is shown in Figures 1B,C. Directly below the cluster a volume with slightly reduced cytosolic concentrations is flanked by regions with slightly higher cytosolic concentrations (Figure 1C). Both deviations are caused by a continuous exchange of proteins from and into the cluster. A net flux of proteins from the cytosol to the membrane is established at the center of the cluster which is balanced by an opposite flux of proteins at the periphery keeping the total amount of proteins in the cluster constant. Hence, proteins are continuously redistributed to the cluster center to counteract lateral diffusion along the plasma membrane (arrows in Figure 1C) similar to other mass conserved polarity models [40], [15].

### Polarization initially emerges as a single broad cap which narrows over time

The initial emergence of polarity can be effectively studied in terms of a linear stability analysis of our model. To this end we extended a previously developed approach for systems with protein exchange between membrane and cytosol [41] to include the spherical symmetry of the cell and the catalyzed membrane attachment of polarity factors. In a linear stability analysis one introduces a small random perturbation to an unpolarized homogenous state of polarity proteins and calculates the time evolution of this protein distribution. Such perturbations represent small concentration fluctuations in protein levels which arise from the Brownian movement of proteins in the cell [42]. Growth of the perturbation implies that the unpolarized state is unstable and that an inhomogeneous pattern arises.

For control cells the linear stability analysis predicted that small random perturbations of the unpolarized state grow and directly evolve into a unique polarity cluster (Figure S1A, Text S1). This was in contrast to the previously made prediction of multiple emerging clusters [15]. Importantly, the stability analysis also predicted that random perturbations always directly evolve towards a single growing cluster when we modified each individual model parameter over more than two orders of magnitude (Text S1).

In general, two qualitatively different patterns can arise as the growing perturbation reaches a macroscopic size. If the initial perturbation is sufficiently small, the emerging pattern will be determined by the linear stability analysis. Conversely, if the initial perturbation is too large, a random pattern emerges that depends on the initial perturbation. By numerical evaluation of the full polarization dynamics we found that even considerable perturbations with an amplitude of 1% of the corresponding average concentration (Materials and Methods) directly evolved into a single cluster as we varied each model parameter between 1/3 and 3 times its control value. Only for parameter values that were well outside this physiologically relevant range we observed that small perturbations could also evolve into multiple clusters before coalescing into a single polarization site due to competition for limited amounts of proteins similar to previous models (see also [40], [15]). An example for formation of multiple caps is shown in Figure S1B. In summary, our model predicts that small random perturbations robustly induce the direct formation of a single polarity cluster as opposed to coarsening dynamics.

**Figure 2:**
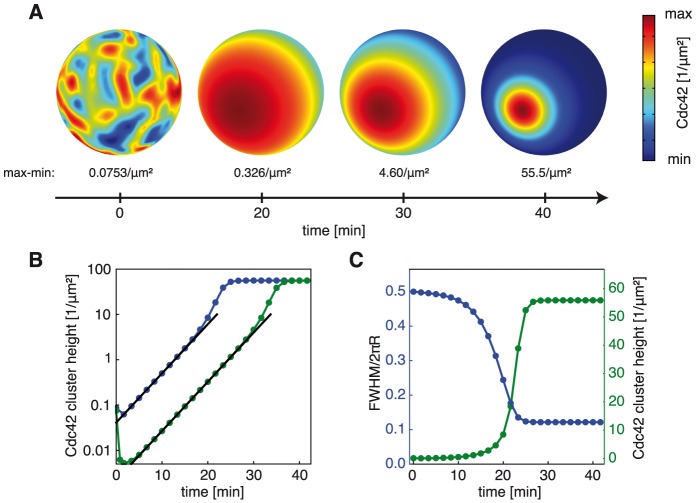
Predicted dynamics of GDI-mediated polarization. **A** Cdc42 density on the plasma membrane for different points in time as obtained from numerical simulations (Movie S1) starting from the unpolarized homogenous solution disturbed by a small random perturbation using control cell parameters. **B** Development of simulated Cdc42 cluster height (maximum density over background) over time starting with a random initial perturbation (green curve) or using a broad cap as initial perturbation (blue curve). The black lines correspond to a growth rate of the corresponding cluster of 0.00417 s^−1^ as predicted from the linear stability analysis. **C** Full width at half maximum (FWHM) of simulated Cdc42 cluster height measured along a great circle through the cap center over circumference 2πR (blue) and simulated Cdc42 cluster height (green) with a broad faint cap as initial perturbation for different points in time.

Given the complex circuitry underlying GDI-mediated polarization we sought to identify characteristic properties of the process which could be verified experimentally. When we examined the temporal evolution of polarization sites in our simulations (Materials and Methods) we could identify two distinct phases. Initially, the random protein distribution was gradually replaced by a single broad cap with exponentially growing amplitude consistent with the prediction from the linear stability analysis (Figure 2A, Figure S1A). In the second phase, the single broad cluster narrowed until it reached a final steady state distribution. To better quantify the narrowing of the cluster we performed simulations with a faint broad cap as an initial perturbation. A single cluster emerged, which showed the same dynamics and shape as those started from random perturbations, confirming that polarization was robust to considerable changes of the initial conditions (Figure 2B). As expected, only the time to reach the final state did depend on the amplitude of the initial perturbation. The time to polarize was consistent with the polarization delay observed after release in synchronized polarization assays indicating that our model provides polarization on physiologically relevant time scales [5]. The numerically obtained cluster shape and growth rate corresponded to the predicted ones from the linear stability analysis (black lines in Figure 2B, Figure S1A). Nonlinear effects enhanced the growth rate when the cluster reached a threshold size (Figure 2B). The time frame where these nonlinear effects are significant coincided with the regime where most of the narrowing of the cluster occurred (Figure 2C). In addition, this was accompanied by most of the absolute increase of the cluster height (Figure 2C). In summary, our model predicts that one characteristic property of direct single site polarization without multiple macroscopic intermediate clusters is the steady narrowing of emerging polarity clusters.

**Figure 3:**
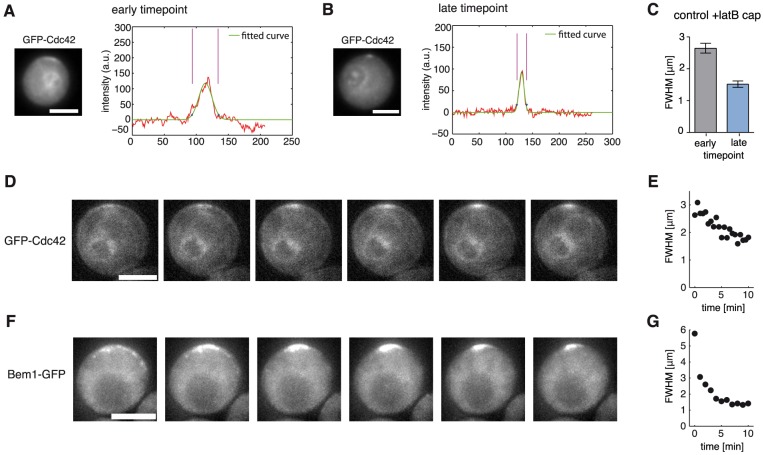
Experimental characterization of GDI-mediated polarization. **A,B** Representative images and cap intensity profiles of latrunculin B-treated control cells expressing GFP-Cdc42 at an early (20–30 min after release, A) and late (30–50 min after release, B) time point during polarization. **C** Comparison of full width at half maximum (FWHM) of GFP-Cdc42 + latrunculin B caps at early (N = 13, SEM = 0.15; 20–30 min after release) and late time points (N = 28, SEM = 0.10; 30–50 min after release), unpaired t-test with Welch correction, p<0.001. **D** Time series showing GFP-Cdc42 cap establishment in a control cell treated with latrunculin B (Movie S2, every second frame starting from frame 12, 30 s time steps between frames). **E** Time evolution of full width at half maximum of GFP-Cdc42 cap shown in (D) and Movie S2 (first width corresponds to frame 10 of Movie S2). **F** Time series showing Bem1-GFP cap establishment in a control cell treated with latrunculin B (Movie S3, every frame starting from frame 8, 60 s time steps between frames). **G** Time evolution of full width at half maximum of Bem1-GFP cap shown in (F) and Movie S3 (first width corresponds to frame 8 of Movie S3). Scale bars: 4 µm.

To validate our prediction in experiments, we released a synchronized population of latrunculin B-treated yeast cells expressing GFP-Cdc42 from G1 arrest and compared their cluster widths at early and late time points (Figure 3A,B). In accordance with our model prediction the clusters were significantly broader shortly after release, compared to the much more focused caps at later points in time (Figure 3C). We also directly monitored the establishment of polarity clusters in latrunculin B-treated cells expressing GFP-Cdc42 or Bem1-GFP. Polarity clusters first emerged as single and faint broad caps, which then progressively became narrower and brighter in the time course of several minutes, in agreement with the model prediction (Figures 2C, 3D–G). Note that the high contrast of Bem1-GFP caps allowed the observation of very early broad-caped states (Figure 3F,G) that were not detectable with the weaker Cdc42 signal (Figure 3D,E). The vast majority of cells directly developed a unique polarization site. Only in 3.8% of 209 latrunculin B-treated cells expressing GFP-Cdc42 we observed intermediate states with multiple clusters during polarization. Hence, our findings suggest that the observed narrowing of clusters is a characteristic signature for the direct emergence of a unique polarization site. The formation of narrow caps did neither depend on the presence of actin structures nor on the formation of a septin diffusion barrier as they also arise in the absence of septin structures [43]. Moreover, our findings suggest that the previously described narrowing of caps in untreated wild-type cells [16] may arise from the dynamics of the GDI-dependent polarization mechanism.

The two-stage process of GDI-mediated polarization identified above allows yeast cells to directly and robustly generate a unique localized Cdc42-GTP cluster, and provides an efficient mechanism to initiate processes downstream of Cdc42 like actin reorganization at a desired position with a minimum risk of transmitting the signal at wrong or multiple positions. Without the direct formation of a single cluster GDI-mediated polarization would instead actively generate several transient clusters with high Cdc42-GTP concentration, and enhance the risk to initiate irreversible processes such as budding at several places on the membrane. For example, the increased occurrence of transient multiple caps in cells strongly overexpressing Bem1 is accompanied by an occasional formation of multiple buds [16].

### Cell cycle induced changes provide temporal control over GDI-mediated polarization

We next asked how changes of system parameters influence GDI-mediated polarization. We focused on parameters, which were experimentally accessible and which directly affected the regulation of Cdc42, such as the rates of GTP hydrolysis and membrane extraction rates of Cdc42 as well as concentrations of Cdc24 and Bem1. Note that we limited our study to moderately increased protein concentrations in order to avoid the generation of unspecific protein-protein interactions that might occur at non-physiological densities.

**Figure 4:**
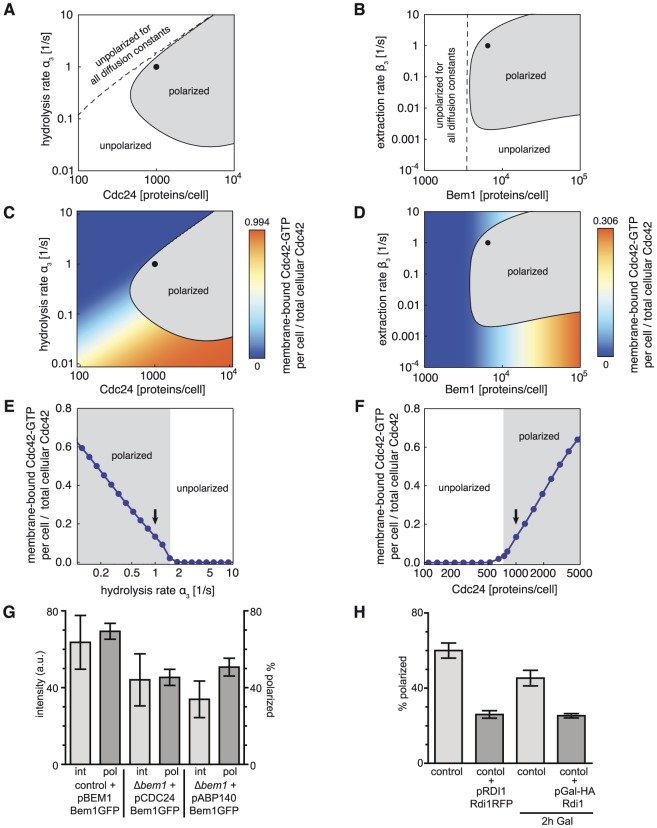
Parameters affecting polarization efficiency and Cdc42 activation. **A,B** Polarization capability predicted from the linear stability analysis shown for different concentrations of the GEF Cdc24 and Cdc42 hydrolysis rates (A) as well as for different concentrations of Bem1 protein and Cdc42 extraction rates (B). In addition, parameter regions are shown where polarization cannot be rescued by a change of the diffusion constants D_2_, D_3_. Black dots indicate values in control cells. **C,D** Plots as in (A, B) but in addition indicating concentration of membrane-bound Cdc42-GTP (as fraction of total cellular Cdc42) in unpolarized cells. Black dots indicate values in control cells. **E,F** Concentration of membrane-bound Cdc42-GTP for different Cdc42 hydrolysis rates (E) and Cdc24 protein numbers per cell (F). The arrows indicate the control cell values. **G** Comparison of average intensity and polarization efficiency of latrunculin B-treated control and Δ*bem1* cells expressing Bem1-GFP from the endogenous or the weak Cdc24 or Abp140 promoter. ANOVA, F(2,6) = 25.52: P = 0.0012, Dunnett's posthoc: control-p24: p<0.001, control-pAbp140: p<0.01. **H** Polarization efficiency of GFP-Cdc42 control cells treated with latrunculin B without or with the overexpression of Rdi1. Rdi1 was either overexpressed as an additional copy under its endogenous promoter or under the Gal-promoter. Unpaired t-test with Welch correction: control - pRDI1-Rdi1RFP: p<0.001, control - pGal-Rdi1: p = 0.015.

Our analysis showed that reduced values for any of the chosen parameters prevented polarization (Figure 4A,B). Importantly, a distinction can be made with regard to the fold change needed to induce this loss. The polarization efficiency was sensitive to a reduction of Cdc24 or Bem1 expression levels but robust to an increase of both. In contrast, we found that while polarization was sensitive to moderately increased Cdc42 hydrolysis and extraction rates, a high fold reduction of both rates was necessary to cause loss of polarization. These results shed new light on previous observations on polarization of mutants in the absence of actin. Cells expressing non-hydrolysable Cdc42 and cells without the GDI Rdi1 represent limiting cases of cells with low Cdc42 hydrolysis and membrane extraction rates, respectively. As predicted by our model, cells cannot polarize in either case (Figures 4A,B; [6], [8]). Moreover, yeast cells without Bem1 [5], with reduced amounts of available Cdc24 [44], or overexpressing the GAP Bem3 (increased Cdc42 hydrolysis, [45]) were not able to polarize. To further validate our model we experimentally tested the predicted sensitivity of polarization efficiency to changes in Bem1 expression or Cdc42 membrane extraction (Figure 4B). In agreement with the model prediction we found polarization efficiency to be sensitive to even small reduction of Bem1 levels, when expressing Bem1 from the weak Cdc24 or Abp140 promoters in latrunculin B-treated cells (Figure 4G). In addition, we studied polarization of yeast cells overexpressing the GDI Rdi1 in the presence of latrunculin B. Even moderate overexpression of Rdi1 resulted in reduced polarization efficiency, in line with the prediction of our model (Figure 4H). Hence, our model provides an accurate description of various mutant phenotypes, which arise from disturbed GDI-mediated polarization.

An important feature emerging from our simulations is the ability of yeast cells to regulate polarization in a switch-like manner when crossing a threshold in Cdc24 concentration or Cdc42 hydrolysis rate (Figure 4A). These two parameters are also regulated during the yeast cell cycle: On the one hand, Cdc24 is sequestered in the nucleus in unpolarized G1 cells and released into the cytosol at the G1-S transition [44], [46]. Inhibition of this relocation process leads to inhibition of cell polarization. On the other hand, reduction of Cdc42 hydrolysis at the G1-S transition by phosphorylation of the Cdc42 GAPs Bem2 and Bem3 has been shown to be crucial for polarization [45]. Hence, our results indicate that GDI-mediated polarization is switched on during the cell cycle by a simultaneous increase in GEF concentrations and reduction of the Cdc42 hydrolysis rate. Given these findings, we wanted to better understand the choice of parameters in control cells and the cell cycle-dependent regulation of polarity. In the following sections we therefore studied the features defining polarized and unpolarized states in more detail.

### Enhancing the positive feedback loops provides simultaneous switch-like regulation of polarization and Cdc42 activation

**Figure 5:**
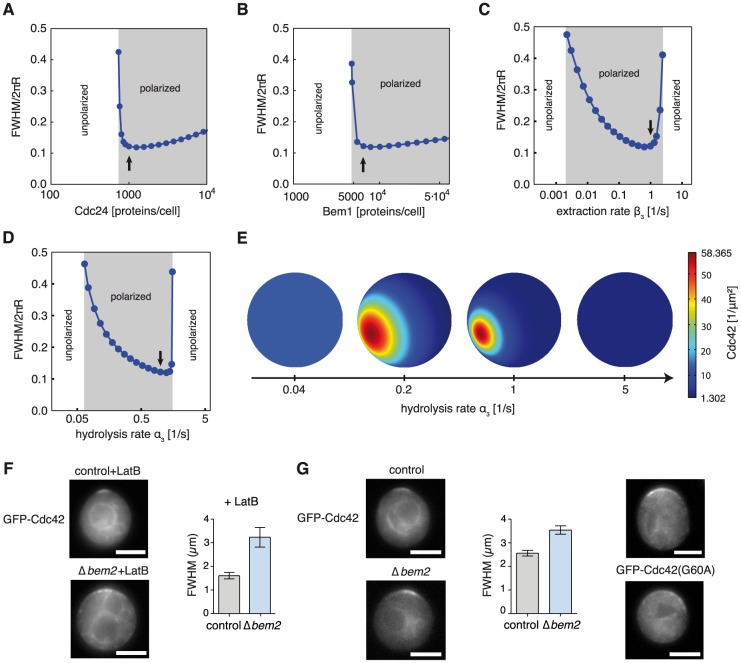
Cluster widths for changing parameters and experimental verification. **A–D** Full width at half maximum of Cdc42 density as obtained in Figure 2 for different Cdc24 (A) and Bem1 (B) concentrations, as well as for different Cdc42 extraction (C) and hydrolysis (D) rates. Arrows indicate the corresponding control cell values. **E** Examples of Cdc42 cluster density calculated for different hydrolysis rates. **F, G** Images and quantification of GFP-Cdc42 cap widths in control and Δ*bem2* cells in the presence (F, unpaired t-test with Welch correction, p<0.001) or absence (G, unpaired t-test with Welch correction, p = 0.01) of latrunculin B. Broad caps are also seen in cells expressing a slowly hydrolyzing GFP-Cdc42^G60A^ mutant. Images and cap widths were acquired 30–50 min after release from G1 arrest. Bar graphs correspond to mean ± SEM. Scale bars: 4 µm.

To reveal the mechanism for how GDI-dependent polarization is disabled we next investigated the membrane-cytosol ratio of Cdc42 in unpolarized cells for different parameter regimes and asked whether failure to polarize is correlated with loss of active Cdc42 on the cell membrane. Interestingly, we found that the regimes with significant amounts of active Cdc42 overlapped but did not coincide with the polarization regimes identified above (Figure 4C,D). For hydrolysis rates much lower and GEF concentrations much higher than the control cell values, there were significant amounts of active Cdc42 even in cells which were unable to polarize (Figure 4C). In this regime the positive feedback loops were still active but the protein transport to the membrane was too slow to counter lateral diffusion on the plasma membrane, and therefore unable to cause cap formation. Consistent with these results, non-hydrolysable Cdc42 has been shown to accumulate on the plasma membrane but is unable to polarize through the GDI-mediated polarization mechanism (Figure 4C; [5]). A similar behavior is found for increasing Bem1 concentrations at low extraction rates (Figure 4D).

These findings stand in contrast to the behavior close to the control cell values at the upper boundary of the polarization regime, where cell cycle induced parameter changes switch cells into an unpolarized state with very low Cdc42-GTP levels (Figure 4C,D). To further analyze the cell's properties at this boundary we calculated the final concentration of active Cdc42 for changing Cdc24 expression levels and Cdc42 hydrolysis rates in the vicinity of the control cell values (Figure 4E,F). Our results show that the production of active Cdc42 is almost completely switched off when polarization breaks down. Hence, cell cycle induced changes simultaneously regulate polarity and the ability for Cdc42-GTP production in a switch-like manner. The GDI-mediated polarization mechanism not only allows selecting a spatially restricted region for Cdc42 to initiate further downstream processes, it also controls the transmission of the Cdc42 signal itself. This switch-like response is genuinely different from the response in a hypothetical system without feedback loops. In the latter case, instead of an abrupt change at a threshold, one would expect the amount of active Cdc42 to gradually increase with the activator concentration (and decrease with the hydrolysis rate) over the whole parameter range such that much higher changes of regulators would be necessary to effectively suppress Cdc42 activity in unpolarized cells. To better understand the origin of the observed switch-like behavior we systematically varied several system parameters, which we assumed to be essential for the pattern formation process. We have previously shown that polarity clusters are maintained by continuous redistribution of polarity factors to counteract the lateral membrane diffusion. Therefore, we expected that loss of polarity might be rescued by a change of the diffusion constants. However, for large parts of the parameter regions considered above we found that loss of polarization could not be rescued in this way (Figure 4A,B). This indicates that loss of polarization in these regimes depends on the nonlinear dynamics of the protein reaction network itself and not solely on insufficient redistribution of proteins. Notably, the diffusion-insensitive parameter regions also coincided with the loss of almost all Cdc42-GTP on the membrane, compare with Figures 4C,D. Hence, we concluded that in these regimes the positive feedback loops of Cdc42 activation and membrane attachment fail and can therefore no longer locally enhance the accumulation of Cdc42-GTP on the membrane as needed for pattern formation. These results provide a unified mechanistic understanding of several mutants causing loss of polarity: Reduced GEF or Bem1 concentrations or enhanced Cdc42 hydrolysis weaken the positive feedback loops of Cdc42 production on the membrane until they fail to operate.

In summary, the GDI-mediated polarization mechanism provides yeast cells with the ability to establish a polarized Cdc42 cluster at a specific membrane position, and at the same time allows an immediate change into an unpolarized state via a ‘feedback switch’ where Cdc42 signaling is suppressed.

### Cell cycle regulated temporal control induces a direct and robust change into highly localized polarization

To shed more light on the consequences of the control cell parameter choice, we studied the final shape of polarity clusters under varying system parameters. Starting from unpolarized cells we numerically calculated the polarization dynamics and subsequently determined the final Cdc42 cluster width for different Cdc24 and Bem1 concentrations as well as different Cdc42 extraction and hydrolysis rates. When we increased Bem1 or Cdc24 concentrations towards control cell values we observed that a narrow final steady state cluster was directly produced as polarization set in (Figure 5A,B). In contrast, if Cdc42 extraction or hydrolysis rates were increased, polarized steady state clusters were initially very broad and only gradually became narrower (Figure 5C,D). However, these narrow final clusters were lost when the rates were increased only slightly beyond the control cell values (Figure 5C,D). Remarkably, the control cell values estimated from experiments were all situated in parameter regions that resulted in almost optimal narrow cluster width (arrows in Figure 5A–D). In summary, we find that the cell cycle dependent concomitant increase of cytosolic Cdc24 and decrease of GAP activity allows yeast cells to directly and robustly switch from an unpolarized state into a highly polarized state, thus ensuring the emergence of a narrow polarity cluster.

One quantitative prediction of our model is that a reduction of the Cdc42 hydrolysis or extraction rates would cause polarity clusters to become less focused compared to the control cell situation (Figure 5C,D). Cdc42 is then expected to spend more time on the plasma membrane and to diffuse over longer distances before Rdi1 extracts it into the cytosol. Numerical solutions of our mechanistic model show the Cdc42 concentration profile on the plasma membrane expected for different hydrolysis rates (Figure 5E). Very low hydrolysis rates were only able to sustain patterns much larger than a yeast cell and therefore resulted in unpolarized cells. With increasing hydrolysis rates initially broad caps appeared that spanned the whole cell. Upon further increase of hydrolysis caps became progressively narrower until a critical value was reached above which polarization disappeared. To verify the predicted broad clusters at low hydrolysis rates we used a yeast strain where the Cdc42 GAP Bem2 was deleted. We then treated these cells with latrunculin B and compared the width of the formed polar caps with those in similarly treated control cells. As predicted, Δ*bem2* cells formed significantly broader caps with nearly twice the width of those in control cells (Figure 5F). A slightly weaker effect on cap width upon *bem2* deletion was also observed in cells not treated with latrunculin, suggesting that GDI-mediated polarization also contributes to the broadening of caps in the presence of actin-mediated Cdc42 recycling (Figure 5G). In addition, the G60A mutant of Cdc42, which exhibits intrinsically reduced hydrolysis rates [47], also formed broader caps, which is consistent with our prediction on Cdc42 polarization with reduced GTP hydrolysis rates (Figure 5G).

These results show that the cell cycle induced reinforcement of the positive feedback loops enables yeast cells to directly and robustly form a highly localized cluster out of an unpolarized state with only slight changes in parameter values. The finding that reduced hydrolysis broadens polarity clusters suggests that the polarity reaction network is optimized by rapid GTPase cycling to achieve maximally focused polarization. This focusing in turn might help to optimally organize morphogenetic processes downstream of Cdc42, such as actin reorganization or septin ring formation [7], [43].

## Discussion

Our results indicate that deterministic reaction-diffusion dynamics provide precise spatial and temporal control of Cdc42-GTP production during yeast cell polarization. The mechanistic model introduced in this work belongs to the class of reaction-diffusion driven pattern-forming systems, and robustly provides direct emergence of a unique polarization site with characteristic narrowing dynamics as confirmed by our experiments. In contrast to our findings, a previous model predicted GDI-mediated polarization via multiple transient competing clusters [15]. However, such polarization dynamics would increase the risk to accidentally initiate downstream signaling through Cdc42 at several places before competition is finished, e.g. budding at several places on the membrane. Our findings suggest the following function of the GDI-mediated polarization pathway: It directly forms a unique polarization site and thereby counteracts the formation of multiple clusters, which might arise from actin-dependent polarization [6], [14], strong particle fluctuations, or due to protein interaction with spatial cues. This does not exclude the occasional emergence of transient multiple clusters at random membrane positions [16], [13]. However, our findings show that the GDI-mediated polarization mechanism itself does not actively form multiple clusters but, in contrast, acts to suppress them and hence reduces the risk of misguided Cdc42 signaling in yeast.

Furthermore, we have shown that enhancement of the positive feedback loops is the key mechanism that initiates polarization. Its characteristic feature is a switch-like change from an unpolarized state to a highly localized polarization site. For example, slight changes in Cdc24 or Bem1 protein concentration are sufficient to directly and robustly induce the formation of a narrow final polarity cluster. The origin of this switch lies in an abruptly gained or lost ability of the positive feedback loops to maintain a state with large amounts of proteins on the membrane, and is not related to the ability to counteract lateral membrane diffusion. In addition, the switch to the polarized state is accompanied by a strong increase of activated Cdc42 levels.

Both release of the GEF Cdc24 from the nucleus and reduction of Cdc42 hydrolysis by phosphorylation of GAPs have been shown to contribute to activation of Cdc42 and subsequent polarity establishment [45], [46], [44]. Our results provide strong evidence that these changes are used for temporal control of GDI-mediated cell polarity as both parameter changes can induce polarization by enhancement of the positive feedback loops. Hence, our findings give a mechanistic understanding of how polarity establishment in yeast is regulated by the cell cycle. The GDI-mediated polarization mechanism fulfills a two-fold function. On the one hand, it generates a highly polarized cluster of Cdc42-GTP on the membrane. On the other hand, it is characterized by a strong decrease of active Cdc42 levels in unpolarized cells compared to polarized cells, meaning that Cdc42 is effectively kept inactive independent of the exact choice of hydrolysis rates and GEF concentrations in unpolarized cells. Thereby yeast cells acquire the ability to initiate Cdc42 signaling in a controlled switch-like manner in a spatially confined region of the plasma membrane. This has to be distinguished from other perturbations of the system where a loss of GDI-mediated polarity does not necessarily abolish Cdc42 signaling. For example, within our model we predict that a reduction of the Cdc42 membrane extraction rate (e.g. by deletion of the GDI RDI1) induces a loss of GDI-mediated polarity but still allows the production of significant amounts of active Cdc42 on the plasma membrane. This is reaffirmed by recent experiments with Δ*rdi1* cells which cannot polarize via the GDI-dependent pathway but still produce sufficient amounts of active Cdc42 to trigger polarization via actin structures [8]. In light of our findings it would be interesting to determine both Cdc42 activity and GDI-dependent polarity establishment in different mutant yeast strains with high temporal resolution. Cells with active Cdc42 and disturbed GDI-mediated polarization are then expected to be prone to the formation of multiple buds. Our model also predicted that polarity clusters would broaden if the Cdc42 extraction or hydrolysis rates were reduced. Consistently, we found that deletion of the GAP Bem2 led to significant broader clusters in latrunculin-treated cells indicating that the biochemical reaction network is indeed adjusted to robustly provide a narrow final cluster.

By combining all results of our study we find that the following key features characterize the polarization process under physiological conditions: (i) GDI-mediated polarity operates in a parameter regime where polarization is accompanied by a significant fold change in Cdc42-GTP amounts. (ii) The pattern-forming mechanism directly produces a single cluster and not several intermediate clusters which then merge into a single cluster. (iii) Cell cycle induced changes in Cdc42 activation directly lead to the robust formation of a narrow final cluster with only very small changes in Cdc24 concentrations or hydrolysis rates. Our results indicate that under typical physiological conditions in yeast cells all of the listed features enable precise control of Cdc42 signaling with a minimized risk of accidentally initiating further processes downstream of Cdc42 at the wrong time or position.

The precise control of Cdc42 signaling induced by the aforementioned features provides evidence for the GEF-mediated positive feedback loops of Cdc42 activation [10] and membrane attachment [21], [36], [14] to be the key molecular mechanisms of GDI-mediated cell polarization suggesting a new perspective on how cell polarity can be established with a minimum set of polarity factors. Our modeling approach for spherical cells can also be extended to other geometries to study pattern formation in similar systems as for example for Min-protein oscillations in *Escherichia coli* [48].

Given the high evolutionary conservation of all involved players we expect GDI-mediated polarization throughout the animal kingdom to show a similar behavior as in yeast. The characteristic finding that reducing the hydrolysis of the polarity GTPase broadens polarity clusters in yeast provides an easily testable prediction to identify similar polarity mechanisms in other eukaryotes. As one example, deletion of the Rop (plant Rho GTPase) GAP Ren-1 defocused polarization of GTPase ROP1 in *Arabidopsis thaliana* pollen tubes [49].

## Materials and Methods

### Reaction-diffusion equations

The set of partial differential equations describing the time evolution of protein concentrations given in the main text reads in spherical coordinates *r*, *θ*, *φ*

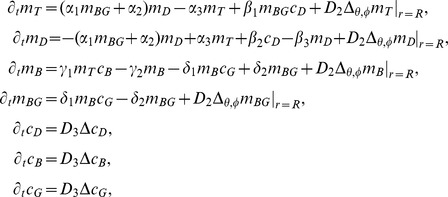
where *Δ_θ,φ_* stands for the angular part of the spherical Laplace operator *Δ*. The membrane concentrations of Cdc42-GTP, Cdc42-GDP, Bem1, and Bem1-Cdc24 complexes at radial position *r = R* are denoted by *m_T_*, *m_D_*, *m_B_*, and *m_BG_*, whereas *c_D_*, *c_B_*, and *c_G_* describe the cytosolic concentrations of Cdc42-GDP, Bem1, and Cdc24. Membrane and cytosolic concentrations are defined as proteins per membrane area (in µm^−2^) and volume (in µm^−3^), respectively. The cytosolic flux of proteins to the membrane is facilitated by the boundary conditions
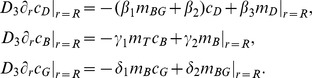



The determined control cell parameters are *D_2_* = 0.03 µm^2^ s^−1^, *D_3_* = 11 µm^2^ s^−1^, *R* = 3.95 µm, *N_42_* = 3000, *N_B_* = 6500, *N_24_* = 1000, *α_1_* = 0.2 µm^2^ s^−1^, *α_2_* = 0.12 min^−1^ , *α_3_* = 1 s^−1^, *β_1_* = 0.266 µm^3^ s^−1^, *β_2_* = 0.28 µms^−1^, *β_3_* = 1 s^−1^, *γ_1_* = 0.2667 µm^3^ s^−1^, *γ_2_* = 0.35 s^−1^, *δ_1_* = 0.00297 µm^3^ s^−1^, and *δ_2_* = 0.35 s^−1^. Note that the protein numbers *N_42_, N_B_, and N_24_* are incorporated via the initial unpolarized concentrations and do not change over time due to protein number conservation.

### Determination of reaction rates

The detachment rates of Bem1 and Cdc24, *γ_2_* and *δ_2_*, were estimated with 0.35 s^−1^ using fluorescence recovery after photobleaching (FRAP) experiments [14] and the intrinsic nucleotide-exchange rate *α_2_* = 0.12 min^−1^ was taken from literature [32]. The Cdc42 FRAP rate of 0.28 s^−1^ [14], [8] provides a lower bound for the Cdc42 hydrolysis and extraction rates, given that Cdc42 needs to be hydrolyzed before it gets extracted by Rdi1. We estimated the hydrolysis and extraction rates α_3_ and β_3_ to 1s^−1^. To fit our model we used an estimate for the fraction of Cdc42 present in the cluster of 10–20% taken from literature [8]. Given that 50–70% of all Cdc42 are found in the inner compartments of the cell [8], we estimated the amount of Cdc42 on the plasma membrane outside the cluster to be equal to the amount inside the cluster. The fractions of Bem1 and Cdc24 in the cluster were estimated to 50% and 10%, respectively, based on fluorescence recovery experiments [5]. As ∼10% of on average 1000 Cdc24 molecules are found in the polarity cluster, which occupies roughly 10% of the membrane [8], we expect an average Cdc24 density of ∼5 µm^−2^ within the cluster. Based on this estimate we chose a GEF-dependent nucleotide-exchange rate *α_1_*, which provides an effective nucleotide-exchange rate of 1 s^−1^ on the same order as hydrolysis and extraction rates in a cluster. The remaining attachment rates *β_1_*, *β_2_*, *γ_1_*, and *δ_1_* were then chosen to approximately achieve fractions of 10% Cdc24, 50% Bem1, and 30% Cdc42 on the membrane of polarized cells based on the estimates given above.

### Simulations

Simulations were performed using Comsol Multiphysics 3.5a.

To generate random initial perturbations we took a random number from the interval [−1,1] for each site of a cubic lattice in space with a spacing of 1 µm. This lattice was then used to produce a continuous perturbation function f(x,y,z) using Comsol's interpolation routine to define functions from a set of random data points. We assumed that pattern formation in our simulations is initiated by small random perturbations with an amplitude of ∼1% of the corresponding unpolarized protein concentration. The perturbations were generated by multiplying the initial unpolarized concentration of all membrane-bound proteins with (1+0.01*f(x,y,z)). Broad caps as initial perturbations were generated by adding a function with linear dependence on one spatial direction to the unpolarized initial concentration of all membrane-bound proteins assuming that the function changes sign at the center of the cell.

### Strain constructions and growth conditions

Techniques for yeast cell culture and genetics were performed as described previously [50]. All yeast strains are described in Table S1. The genotypes of the yeast strains are as follows: MATa cln1::HisG cln2Δ cln3::HisG YipLac204-MET-CLN2::TRP1 ura3 his3-11.15 ade2-1 can1-100 (gift from M. Peter). For the polarization assay logarithmically growing cyclin-depleted cells (in SC-Methionine and 2% glucose medium) were arrested in G1 by growth for 4 h in SC-all medium supplemented with 3 mM Methionine. LatB was added at 400 µM with release. For time-lapse microscopy cells were transferred to glass bottom dishes (MatTek) 1 h prior to G1 release.

For Gal-induction experiments, Rdi1 from their endogenous promoter or a galactose-inducible promoter cells were grown overnight in SC-methionine and 2% glucose, washed three times with distilled water, diluted into SC-methionine containing 2% raffinose, and then arrested for 3 h in G1 in Sc-all supplemented with 2% raffinose and 3 mM methionine. Cells expressing Rdi1 from the *GAL* promoter were induced for 2 h by addition of 2% galactose. Cells were released from G1 arrest in the presence of LatB and polarization efficiency was determined after 60 min.

### Plasmid constructions and genomic tagging

pRS306 and pRS315 were used for N-terminal plasmid construction (Table S2). All primers used within this study are listed in Table S3.

### Microscopy and imaging

Coverslips or glass bottom dishes were coated with 5 µl 2 mg/ml ConA (Sigma) prior to sample observation. Raw images were used for quantifications and analyses. Depicted images were background-subtracted and light- and contrast-optimized for better visualization. Single pictures of cells were taken on a Zeiss Imager A1 upright microscope system with an Olympus 1.3NA 100× Objective Lense, an X-Cite Halogen lamp from Visitron Systems, and an AndoriXON EM CCD Camera. Images were acquired with Metamorph 7.O software. Time lapse movies of Bem1-GFP were acquired on a custom TIRF setup from Till photonics based on a fully automated iMic-stand with 1.45 NA 100× objective from Olympus. The 488 nm (Coherent Saphire) laser was directed through an AOTF and directly coupled into the iMic. A galvanometer-driven 2-axis scan head was used to adjust the TIRF angle and an additional galvanometer was used to switch between regular fluorescence and TIRF mode. Images were collected with a cooled AndoriXON DU-897 EM CCD camera. Acquisition was controlled by the Live-Acquisition software package. Oblique illumination [51] was used for time lapse microscopy. Acquisition of Bem1-GFP cells was taken with 60 ms exposure, 60 s frame rate and 60% laser power and maximum projection of 3 z-stacks, increment 0.4 µm. Cells expressing GFP-Cdc42 were arrested, released and latrunculin-treated in an ONIX (CellASIC) microfluidics device. Acquisition of GFP-Cdc42 cells was taken with 30 ms exposure, 30 s frame rate, 40% laser power, and maximum projection of 3 z-stacks, increment 0.2 µm.

### Intensity measurements

Control cells expressing Bem1 from its endogenous promoter or *bem1*Δ cells expressing Bem1 from the Cdc24 or Abp140 promoter where arrested in G1 and average intensities (in homogenously strained regions) were measured using Fiji-software.

### Image processing

For visualization purposes all cell images/movies were background-subtracted, contrast-enhanced and a Gaussian blur filter was used to smooth the images. All analyses were done on raw images.

### Experimental cap width determination

Microscopy images of the cell equator (with cap located on the equator) were used to determine cap expansions. A line scan was manually drawn along the cell membrane (including the cap). For automatic analysis of the caps used for Figures 3C, 5F,G the raw images were processed with a Gaussian blur with a 3×3 matrix to mimic average intensities along the line scan. Intensities along the line scan were retrieved for each position on the cell membrane. A Gaussian was then fitted on the intensities and the cap width was determined as the full width at half maximum.

## Supporting Information

Figure S1 **Details of polarization dynamics.** **A** Plot of the growth rate w_l,m_ determined from the linear stability analysis using control cell parameters for different modes *l*. The first mode corresponds to a growing single cluster whereas the higher modes decay. **B** Numerically obtained relative Cdc42 protein concentration for different time points of a polarizing cell with a Cdc24 protein number 10 times its control cell value. The initial perturbation was generated as for control cells and is described in the Materials and Methods section.
(PDF)

Movie S1 **Numerically obtained Cdc42 polarization dynamics.** Movie showing time evolution of numerically obtained Cdc42 density on the membrane for control cell values with 2 min time step between frames. Density measured in µm^−2^.
(MP4)

Movie S2 **GFP-Cdc42 cap establishment.** Movie showing GFP-Cdc42 cap establishment in a control cell treated with latrunculin B with 30 s time steps between frames.
(MP4)

Movie S3 **Bem1-GFP cap establishment.** Movie showing Bem1-GFP cap establishment in a control cell treated with latrunculin B with 60 s time steps between frames.
(MP4)

Table S1 **Yeast strains.**
(PDF)

Table S2 **Plasmids.**
(PDF)

Table S3 **Primers.**
(PDF)

Text S1 **Supplemental materials and methods.**
(PDF)
