# Supplementary figures and images for: GDI-Mediated Cell Polarization in Yeast Provides Precise Spatial and Temporal Control of Cdc42 Signaling
Source: PLoS Comput Biol. 2013 Dec 12;9(12):e1003396. doi: 10.1371/journal.pcbi.1003396 (PMC3861033; doi:10.1371/journal.pcbi.1003396)

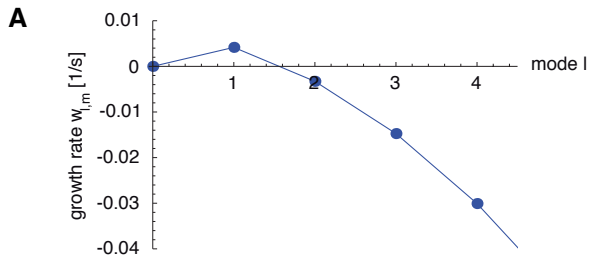

**B**

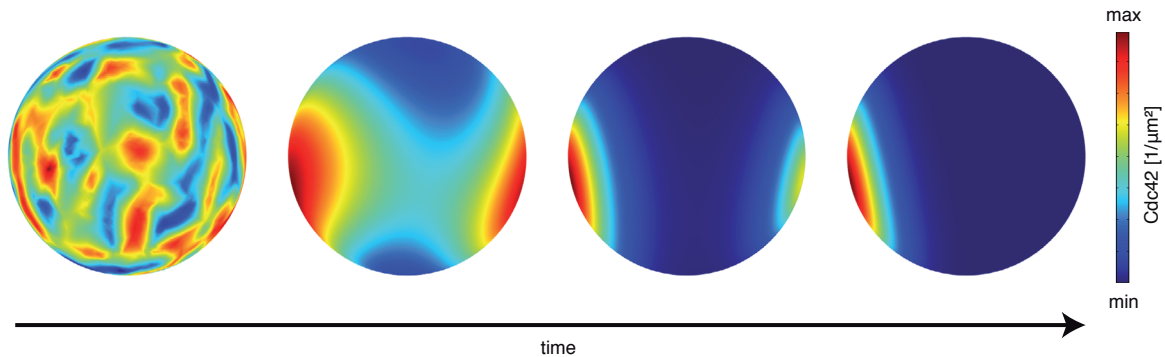

Supplement: Figure S1 — Details of polarization dynamics. A Plot of the growth rate wl,m determined from the linear stability analysis using control cell parameters for different modes l. The first mode corresponds to a growing single cluster whereas the higher modes decay. B Numerically obtained relative Cdc42 protein concentration for different time points of a polarizing cell with a Cdc24 protein number 10 times its control cell value. The initial perturbation was generated as for control cells and is described in the Materials and Methods section. (PDF) [file pcbi.1003396.s001.pdf]
