# Supplementary material for: GDI-Mediated Cell Polarization in Yeast Provides Precise Spatial and Temporal Control of Cdc42 Signaling
Source: PLoS Comput Biol. 2013 Dec 12;9(12):e1003396. doi: 10.1371/journal.pcbi.1003396 (PMC3861033; doi:10.1371/journal.pcbi.1003396)
Supplement: Table S1 — Yeast strains. (PDF) [file pcbi.1003396.s005.pdf]

Table S1: Yeast strains

Strain	Genotype*	Source/Reference
RWS116	MATa *cln1*::HisG *cln2*Δ *cln3*::HisG YipLac204-MET-CLN2::TRP1 *ura3 his3-11.15 ade2-1 can1-100*	[1]
RWS1028	Δ*bem2*::G418	[2]
RWS1035	Δ*bem2*::G418 pCDC42-myc-GFP-CDC42::URA3 (RWC108)	[2]
RWS1421	pCDC42-myc-GFP-CDC42::URA3 (RWC108)	[2]
RWS1045	pBem1-Bem1-GFP::URA3 (RWC138)	This study
RWS 147	pGal-HA-RDI1::LEU pCDC42-myc-GFP-CDC42::LEU	This study
RWS 1758	pRDI1-RDI1-mRFPRuby::LEU (RWC 856) pCDC42-myc-GFP-CDC42::URA3 (RWC 108)	This study
RWS 1739	Δ*bem1*::G418 pABP140-BEM1-GFP::LEU (RWC 899)	This study
RWS 1740	Δ*bem1*::G418 pCDC24-BEM1-GFP::LEU (RWC 900)	This study
RWS 1036	pCDC42-myc-GFP-CDC42G60A::URA3	[2]

*all strains were made in RWS 116 background

References

1. Gulli MP, Jaquenoud M, Shimada Y, Niederhäuser G, Wiget P, et al. (2000) Phosphorylation of the Cdc42 exchange factor Cdc24 by the PAK-like kinase Cla4 may regulate polarized growth in yeast. Mol Cell 6: 1155-1167
2. Freisinger T, Klünder B, Johnson J, Müller N, Pichler G, et al. (2013) Establishment of a robust single axis of cell polarity by coupling multiple positive feedback loops. Nat Commun 4: 1807
