# Supplementary material for: GDI-Mediated Cell Polarization in Yeast Provides Precise Spatial and Temporal Control of Cdc42 Signaling
Source: PLoS Comput Biol. 2013 Dec 12;9(12):e1003396. doi: 10.1371/journal.pcbi.1003396 (PMC3861033; doi:10.1371/journal.pcbi.1003396)
Supplement: Table S2 — Plasmids. (PDF) [file pcbi.1003396.s006.pdf]

Table S2: Plasmids

Plasmid	Origin	Description	Source/Reference
RWC108	pRS306	GFP-CDC42 under the control of the endogenous CDC42-promoter in a pRS306 backbone for integration into the URA3 locus after linearization	[1]
RWC138	pRS306	BEM1-GFP under the control of the endogenous BEM1-promoter in a pRS306 backbone for integration into the URA3 locus after linearization	Gift from Mathias Peter
RWC856	pRS315	RDI1-mRFPRuby under the control of the endogenous RDI1-promoter in a pRS315 backbone LEU-CEN plasmid	This study
RWC 899	pRS315	BEM1-GFP under the control of the endogenous pABP140-promoter in a pRS315 backbone LEU-CEN plasmid	This study
RWC 900	pRS315	BEM1-GFP under the control of the endogenous pCDC24-promoter in a pRS315 backbone LEU-CEN plasmid	This study

References

1. Wedlich-Soldner R, Wai SC, Schmidt T, Li R (2004) Robust cell polarity is a dynamic state established by coupling transport and GTPase signaling. J Cell Biol 166: 889-900
