# Supplementary material for: GDI-Mediated Cell Polarization in Yeast Provides Precise Spatial and Temporal Control of Cdc42 Signaling
Source: PLoS Comput Biol. 2013 Dec 12;9(12):e1003396. doi: 10.1371/journal.pcbi.1003396 (PMC3861033; doi:10.1371/journal.pcbi.1003396)
Supplement: Table S3 — Primers. (PDF) [file pcbi.1003396.s007.pdf]

Table S3: Primers

Primer	Sequence 5’→3’
RWS347	AGGCAAGAGATCAGGCGGAAAGA
RWS346	AGAAGCAAGCTACGTTGCAGCCA
RWS885	CAGGTTTCATTGGAGGTGC
RWS265	GGTGCTCAACAATTCAGTTCT
RWS352	TGCACCAACATACCGTTTTGC
RWS351	TGATGGTAAATCCCGTCCTGC
RWS48	GTATTCTGGGCCTCCATG
RWS47	GATACTAACGCCGCCATC
