# Supplementary material for: GDI-Mediated Cell Polarization in Yeast Provides Precise Spatial and Temporal Control of Cdc42 Signaling
Source: PLoS Comput Biol. 2013 Dec 12;9(12):e1003396. doi: 10.1371/journal.pcbi.1003396 (PMC3861033; doi:10.1371/journal.pcbi.1003396)
Supplement: Text S1 — Supplemental materials and methods. (PDF) [file pcbi.1003396.s008.pdf]

GDI-mediated cell polarization in yeast provides precise spatial and temporal control of Cdc42 signaling

Ben Klünder[1], Tina Freisinger[2], Roland Wedlich-Söldner[2,3], and Erwin Frey[1]

[1]Arnold Sommerfeld Center for Theoretical Physics (ASC) and Center for NanoScience (CeNS), Department of Physics, Ludwig-Maximilians-Universität München, Theresienstraße 37, D-80333 München, Germany
[2]Max Planck Institute of Biochemistry, Cellular Dynamics and Cell Patterning, Am Klopferspitz 18, D-82152 Martinsried, Germany
[3]Present address: University of Münster, Institute of Cell Dynamics and Imaging, Von-Esmarch-Str. 56, D-48149 Münster, Germany

Contents

Supplemental materials and methods 2

Linear stability analysis 2
Robustness of polarization dynamics 5
References 5

Supplemental materials and methods

Linear stability analysis

The initial dynamics of emerging polarity clusters can be studied using a linear stability analysis for problems with cytosol-membrane coupling [1]. Using this framework we asked how a small perturbation of the spatially homogenous steady state distribution of proteins would evolve in time. A decay of the perturbation implies that the homogenous state is stable and no polarization occurs. The advantage of this approach lies in the use of developed standard methods for systems of linear differential equations. The ability of the system to polarize for a certain set of parameters can be tested by finding the roots of polynomials instead of simulating the full dynamics each time. For all cases tested we found perfect agreement between linear stability analysis and simulation in predicting the spontaneous polarization efficiency. The full set of reaction-diffusion equations from the main text reads in spherical coordinates r,θ,ϕ

$$
\begin{aligned}
\partial_t m_T &= (\alpha_1 m_{BG} + \alpha_2) m_D - \alpha_3 m_T + \beta_1 m_{BG} c_D + D_2 \Delta_{\theta,\phi} m_T \big|_{r=R}, \\
\partial_t m_D &= -(\alpha_1 m_{BG} + \alpha_2) m_D + \alpha_3 m_T + \beta_2 c_D - \beta_3 m_D + D_2 \Delta_{\theta,\phi} m_D \big|_{r=R}, \\
\partial_t m_B &= \gamma_1 m_T c_B - \gamma_2 m_B - \delta_1 m_B c_G + \delta_2 m_{BG} + D_2 \Delta_{\theta,\phi} m_B \big|_{r=R}, \\
\partial_t m_{BG} &= \delta_1 m_B c_G - \delta_2 m_{BG} + D_2 \Delta_{\theta,\phi} m_{BG} \big|_{r=R}, \\
\partial_t c_D &= D_3 \Delta c_D, \\
\partial_t c_B &= D_3 \Delta c_B, \\
\partial_t c_G &= D_3 \Delta c_G,
\end{aligned}
$$

where $\Delta_{\theta,\phi}$ stands for the angular part of the spherical Laplace operator Δ. The diffusive flux to the membrane is incorporated by the boundary conditions

$$
\begin{aligned}
D_3 \partial_r c_D \big|_{r=R} &= -(\beta_1 m_{BG} + \beta_2) c_D + \beta_3 m_D \big|_{r=R}, \\
D_3 \partial_r c_B \big|_{r=R} &= -\gamma_1 m_T c_B + \gamma_2 m_B \big|_{r=R}, \\
D_3 \partial_r c_G \big|_{r=R} &= -\delta_1 m_B c_G + \delta_2 m_{BG} \big|_{r=R}.
\end{aligned}
$$

As long as the perturbation is small compared to the homogenous steady state solution one considers a linearized version of the full set of reaction-diffusion equations as a good approximation for describing the time evolution of perturbations. The linearized version of full reaction-diffusion equations for the perturbations δm_x, δc_x reads

$$\partial_t \delta m_T = (D_2 \Delta_{\theta,\phi} - \alpha_3)\delta m_T + (\alpha_1 m_{BG}^0 + \alpha_2)\delta m_D + (\alpha_1 m_D^0 + \beta_1 c_D^0)\delta m_{BG} + \beta_1 m_{BG}^0 \delta c_D\Big|_{r=R},$$
$$\partial_t \delta m_D = \alpha_3 \delta m_T + (D_2 \Delta_{\theta,\phi} - \alpha_1 m_{BG}^0 - \alpha_2 - \beta_3)\delta m_D - \alpha_1 m_D^0 \delta m_{BG} + \beta_2 \delta c_D\Big|_{r=R},$$
$$\partial_t \delta m_B = \gamma_1 c_B^0 \delta m_T + (D_2 \Delta_{\theta,\phi} - \gamma_2 - \delta_1 c_G^0)\delta m_B + \delta_2 \delta m_{BG} + \gamma_1 m_T^0 \delta c_B - \delta_1 m_B^0 \delta c_G\Big|_{r=R},$$
$$\partial_t \delta m_G = \delta_1 c_G^0 \delta m_B + (D_2 \Delta_{\theta,\phi} - \delta_2)\delta m_{BG} + \delta_1 m_B^0 \delta c_G\Big|_{r=R},$$
$$\partial_t \delta c_D = D_3 \Delta c_D,$$
$$\partial_t \delta c_B = D_3 \Delta c_B,$$
$$\partial_t \delta c_G = D_3 \Delta c_G.$$

For the linearized boundary conditions one gets

$$D_3 \partial_r \delta c_D\Big|_{r=R} = \beta_3 \delta m_D - \beta_1 c_D^0 \delta m_{BG} - (\beta_1 m_{BG}^0 + \beta_2)\delta c_D\Big|_{r=R},$$
$$D_3 \partial_r \delta c_B\Big|_{r=R} = -\gamma_1 c_B^0 \delta m_T + \gamma_2 \delta m_B - \gamma_1 m_T^0 \delta c_B\Big|_{r=R},$$
$$D_3 \partial_r \delta c_G\Big|_{r=R} = -\delta_1 c_G^0 \delta m_B + \delta_2 \delta m_{BG} - \delta_1 m_B^0 \delta c_G\Big|_{r=R}.$$

The constants m_x^0, c_x^0 denote the values of m_x, c_x of the physical spatially homogenous steady state solution of the full set of reaction-diffusion equations. Note that these quantities depend on the particle numbers.

To solve the linearized set of equations we made use of the spherical symmetry. We expanded the perturbations in a series of real spherical harmonics $Y_{l,m}(\theta,\phi)$ [2], assumed an exponential time dependence, and used the ansatz

$$\delta m_x(t,\theta,\phi) = \sum_{l=0}^{\infty} \sum_{m=-l}^{l} \delta m_x^{l,m} Y_{l,m}(\theta,\phi) e^{\omega_{l,m} t},$$
$$\delta c_x(r,t,\theta,\phi) = \sum_{l=0}^{\infty} \sum_{m=-l}^{l} \delta c_x^{l,m} A^{l,m}(r) Y_{l,m}(\theta,\phi) e^{\omega_{l,m} t}.$$

The aim of the following calculation is to find the largest real part $w_{l,m}$ of all rates $\omega_{l,m}$ as a positive value implies a growth of pattern induced by a small perturbation. By combining the expansions of the cytosolic perturbations δc_x with the corresponding diffusion equations for the cytosol and using $r^2 \Delta_{\theta,\phi} Y_{l,m}(\theta,\phi) = -l(l+1) Y_{l,m}(\theta,\phi)$ [3] we arrived at

$$0 = \left[r^2 \partial_r^2 + 2r\partial_r - l(l+1) - \omega_{l,m} r^2 / D_3 \right] A^{l,m}(r)$$

for each mode l,m. Physical solutions of this equation are the modified spherical Bessel functions of the first kind $i_l(r\sqrt{\omega_{l,m} / D_3})$ [4]. For the cytosolic perturbations we got

$$\delta c_x(r,t,\theta,\phi) = \sum_{l=0}^{\infty} \sum_{m=-l}^{l} \delta c_x^{l,m} i_l(r\sqrt{\omega_{l,m} / D_3}) Y_{l,m}(\theta,\phi) e^{\omega_{l,m} t}.$$

By putting this result and the expansion for the membrane perturbations δm_x into the linearized boundary conditions we got for each mode l,m

$$D_3 \delta c_D^{l,m} \partial_r i_l (r\sqrt{\omega_{l,m} / D_3})\Big|_{r=R} = \beta_3 \delta m_D^{l,m} - \beta_1 c_D^0 \delta m_{BG}^{l,m} - (\beta_1 m_{BG}^0 + \beta_2) \delta c_D^{l,m} i_l (R\sqrt{\omega_{l,m} / D_3}),$$
$$D_3 \delta c_B^{l,m} \partial_r i_l (r\sqrt{\omega_{l,m} / D_3})_{r=R} = -\gamma_1 c_B^0 \delta m_T^{l,m} + \gamma_2 \delta m_B^{l,m} - \gamma_1 m_T^0 \delta c_B^{l,m} i_l (R\sqrt{\omega_{l,m} / D_3}),$$
$$D_3 \delta c_G^{l,m} \partial_r i_l (r\sqrt{\omega_{l,m} / D_3})_{r=R} = -\delta_1 c_G^0 \delta m_B^{l,m} + \delta_2 \delta m_{BG}^{l,m} - \delta_1 m_B^0 \delta c_G^{l,m} i_l (R\sqrt{\omega_{l,m} / D_3}).$$

Using these equations we reexpressed the cytosolic perturbations δc_x in terms of the membrane perturbation amplitudes $\delta m_x^{l,m}$ as

$$\delta c_D(r,t,\theta,\phi) = \sum_{l=0}^{\infty} \sum_{m=-l}^{l} \frac{(\beta_3 \delta m_D^{l,m} - \beta_1 c_D^0 \delta m_{BG}^{l,m}) i_l (r\sqrt{\omega_{l,m} / D_3}) Y_{l,m}(\theta,\phi) e^{\omega_{l,m} t}}{D_3 \partial_r i_l (r\sqrt{\omega_{l,m} / D_3})\Big|_{r=R} + (\beta_1 m_{BG}^0 + \beta_2) i_l (R\sqrt{\omega_{l,m} / D_3})},$$
$$\delta c_B(r,t,\theta,\phi) = \sum_{l=0}^{\infty} \sum_{m=-l}^{l} \frac{(-\gamma_1 c_B^0 \delta m_T^{l,m} + \gamma_2 \delta m_B^{l,m}) i_l (r\sqrt{\omega_{l,m} / D_3}) Y_{l,m}(\theta,\phi) e^{\omega_{l,m} t}}{D_3 \partial_r i_l (r\sqrt{\omega_{l,m} / D_3})\Big|_{r=R} + \gamma_1 m_T^0 i_l (R\sqrt{\omega_{l,m} / D_3})},$$
$$\delta c_G(r,t,\theta,\phi) = \sum_{l=0}^{\infty} \sum_{m=-l}^{l} \frac{(-\delta_1 c_G^0 \delta m_B^{l,m} + \delta_2 \delta m_{BG}^{l,m}) i_l (r\sqrt{\omega_{l,m} / D_3}) Y_{l,m}(\theta,\phi) e^{\omega_{l,m} t}}{D_3 \partial_r i_l (r\sqrt{\omega_{l,m} / D_3})\Big|_{r=R} + \delta_1 m_B^0 i_l (R\sqrt{\omega_{l,m} / D_3})}.$$

These expressions allowed us to reduce the linearized set of reaction-diffusion equations to four equations with four independent variables $\delta m_x^{l,m}$. After introducing the abbreviations

$$A(\omega_{l,m}) = \frac{1}{D_3 \partial_r i_l (r\sqrt{\omega_{l,m} / D_3})\Big|_{r=R} / i_l (R\sqrt{\omega_{l,m} / D_3}) + \beta_1 m_{BG}^0 + \beta_2},$$
$$B(\omega_{l,m}) = \frac{1}{D_3 \partial_r i_l (r\sqrt{\omega_{l,m} / D_3})\Big|_{r=R} / i_l (R\sqrt{\omega_{l,m} / D_3}) + \gamma_1 m_T^0},$$
$$C(\omega_{l,m}) = \frac{1}{D_3 \partial_r i_l (r\sqrt{\omega_{l,m} / D_3})\Big|_{r=R} / i_l (R\sqrt{\omega_{l,m} / D_3}) + \delta_1 m_B^0}$$

we combined all results and arrived at

$$\begin{aligned}
0 \;=\;& \left[-D_2 l(l+1)/R^2 - \alpha_3 - \omega_{l,m}\right]\delta m_T^{l,m} + \left[\alpha_1 m_{BG}^0 + \alpha_2 + \beta_1 m_{BG}^0 \beta_3 A(\omega_{l,m})\right]\delta m_D^{l,m} \\
&+ \left\{\alpha_1 m_D^0 + \beta_1 c_D^0 \left[1 - \beta_1 m_{BG}^0 A(\omega_{l,m})\right]\right\}\delta m_{BG}^{l,m}, \\
0 \;=\;& \alpha_3 m_T^{l,m} + \left\{-D_2 l(l+1)/R^2 - \alpha_1 m_{BG}^0 - \alpha_2 - \beta_3\left[1 - \beta_2 A(\omega_{l,m})\right] - \omega_{l,m}\right\}\delta m_D^{l,m} \\
&+ \left[-\alpha_1 m_D^0 - \beta_2 \beta_1 c_D^0 A(\omega_{l,m})\right]\delta m_{BG}^{l,m}, \\
0 \;=\;& \gamma_1 c_B^0 \left[1 - \gamma_1 m_T^0 B(\omega_{l,m})\right]\delta m_T^{l,m} + \left\{-D_2 l(l+1)/R^2 - \gamma_2\left[1 - \gamma_1 m_T^0 B(\omega_{l,m})\right]\right. \\
&\left. - \delta_1 c_G^0 \left[1 - \delta_1 m_B^0 C(\omega_{l,m})\right] - \omega_{l,m}\right\}\delta m_B^{l,m} + \delta_2\left[1 - \delta_1 m_B^0 C(\omega_{l,m})\right]\delta m_{BG}^{l,m}, \\
0 \;=\;& \delta_1 c_G^0 \left[1 - \delta_1 m_B^0 C(\omega_{l,m})\right]\delta m_B^{l,m} + \left\{-D_2 l(l+1)/R^2 - \delta_2\left[1 - \delta_1 m_B^0 C(\omega_{l,m})\right] - \omega_{l,m}\right\}\delta m_{BG}^{l,m}
\end{aligned}$$

for each mode l,m. The rates $\omega_{l,m}$ of nontrivial solutions can be found by setting the determinant of the coefficient matrix of this set of equations equal to zero. The growth rates $w_{l,m}$ we were interested in are the maximum real part of all possible $\omega_{l,m}$ for a certain mode l,m and were calculated using Mathematica 8. Note that

$w_{l,m}$ and $\omega_{l,m}$ only depend on l. The parameters used are given in the main text.

In general, random perturbations will be made of a superposition of all possible modes. Under physiological conditions only the first modes *1,m* have a positive growth rate and the initial shape of emerging wild-type clusters is predicted to be a superposition of the first real spherical harmonics (Figure S1A).

Robustness of polarization dynamics

Next we asked whether the direct evolution of initial perturbations towards a single cluster is a robust property of polarization. We varied each model parameter separately and found that only for large changes of some parameters the linear stability analysis predicts higher modes (l=2,3,4,...) to have the largest growth rate. The changes from the control cell values needed to induce this behavior are 16x for N_{24}, 46x for N_B, 0.031x for D_2, and 86x for δ_1.

However, given that also higher modes have a positive growth rate it is possible that a perturbation reaches a size where nonlinear effects become important before a single mode dominates its shape. This is the case if the initial perturbation is too large for the given differences of growth rates. To address this issue we numerically simulated the full polarization dynamics starting with different realizations of the perturbation function f(x,y,z) defined in the Materials and Methods section. We found that the initial small perturbations still directly evolved into a single cluster as we varied each model parameter separately from 1/3 to 3 times its wild-type value.

References

1. Levine H, Rappel WJ (2005) Membrane-bound Turing patterns. Phys rev E 72: 061912
2. Whittaker ET, Watson GN (1962) A course of modern analysis (4th ed.). Cambridge University Press, USA
3. Triebel H (1982) Analysis und mathematische Physik. Carl Hauser Verlag, München, Germany
4. Abramowitz M, Stegun IA, eds. (1984) Pocketbook of Mathematical Functions. Verlag Harry Deutsch, Frankfurt/Main, Germany
